# Recent advances in earth-abundant transition metal-catalyzed dihydrosilylation of terminal alkynes

**DOI:** 10.3389/fchem.2024.1411140

**Published:** 2024-05-27

**Authors:** Chanmi Lee, Dohun Lee, Sung You Hong, Byunghyuck Jung, Sangwon Seo

**Affiliations:** ^1^ Department of Physics and Chemistry, DGIST, Daegu, Republic of Korea; ^2^ School of Undergraduate Studies, DGIST, Daegu, Republic of Korea; ^3^ Department of Chemistry, UNIST, Ulsan, Republic of Korea

**Keywords:** alkyne functionalization, dihydrosilylation, earth-abundant metal catalysis, organosilanes, sustainable synthesis

## Abstract

Over the past few years, earth-abundant transition metal-catalyzed hydrosilylation has emerged as an ideal strategy for the synthesis of organosilanes. The success in this area of research has expanded to the advancements of alkyne dihydrosilylation reactions, offering broadened synthetic applications through the selective installation of two silyl groups. In particular, catalysts based on Fe, Co, and Ni have engendered enabling platforms for mild transformations with a range of distinct regioselectivity. This mini-review summarizes recent advances in this research field, highlighting the unique features of each system from both synthetic and mechanistic perspectives.

## Introduction

Owing to the importance of organosilanes in broad research fields, the development of general methods for their preparation has garnered significant attention in the past few decades (Roesky, 2017). Among those that engendered considerable efforts, the hydrosilylation of unsaturated C–C bonds has been established as a powerful strategy accommodating 100% atom economy (Marciniec, 2009), in which the addition of a silyl group and hydrogen atom across the π-system gives direct access to organosilanes from readily available alkenes and alkynes. These alluring features called for extensive investigations toward the relevant reaction developments and, consequently, a diverse range of catalytic systems have appeared for hydrosilylation reactions. In particular, the advent of earth-abundant metal-catalyzed methods (Greenhalgh et al., 2015; Sun and Deng, 2016; Chen et al., 2018; Obligacion and Chirik, 2018) has contributed substantially to allaying the cost and environmental issues associated with the conventional strategies that were dominated by precious metal catalysts. The synthetic merits of such advancements can be reflected by their wide applications in the industrial production of organosilicon compounds, with the contemporary catalysts offering the opportunity for chemo-, regio-, and stereocontrol of the reactions.

**SCHEME 1:**
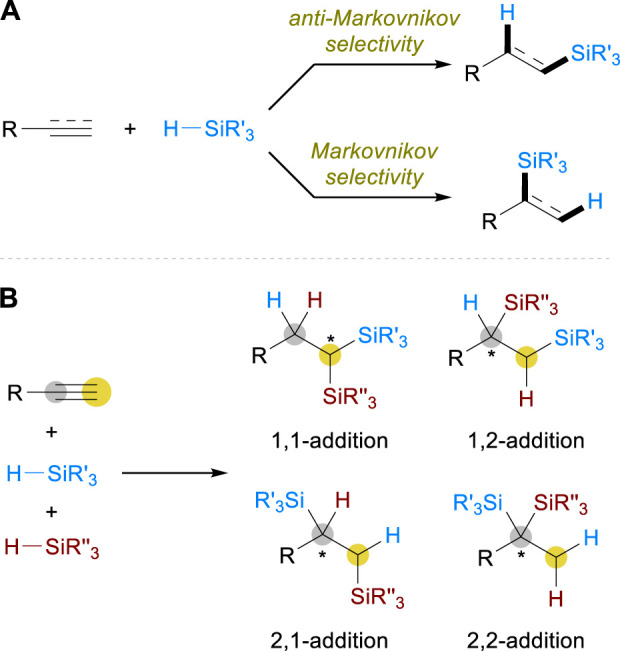
Regio- and stereoselectivity in **(A)** Alkene/alkyne monohydrosilylation and **(B)** Alkyne dihydrosilylation.

Hydrosilylation reactions are generally classified into Markovnikov and anti-Markovnikov hydrosilylation in terms of regiochemistry, the former of which installs a silyl group on the more substituted carbon while the latter inserts it on the less substituted carbon (Scheme 1A). The modification of ligands is often crucial to attain control over the regioselectivity, as well as for the realization of enantioselective reactions where branched silane products are produced to introduce a stereocenter. A large body of studies has been carried out by relying on this common tactic, and the related discoveries of alkene and alkyne hydrosilylation have been discussed intensively in several reviews (Greenhalgh et al., 2015; Nakajima and Shimada, 2015; Sun and Deng, 2016; Zaranek et al., 2016; Chen et al., 2018; Obligacion and Chirik, 2018; de Almeida et al., 2021; He et al., 2022). On the other hand, the selectivity control becomes more complicated and challenging when it comes to the development of alkyne dihydrosilylation, which represents the net addition of two silyl groups and two hydrogen atoms across a C–C triple bond (Scheme 1B). The possible regiochemistry includes 1,1-, 1,2-, 2,1-, and 2,2-additions of silanes, and the control of stereochemistry should also be considered in all cases especially if two different silane sources were to be inserted. These challenges in selectivity control impeded the advances of such transformations in some ways and, as a result, only a few limited examples were reported (Shimada et al., 2002) until the recent emergence of earth-abundant metal-catalyzed strategies. This mini-review covers the recent advances in this field, specifically highlighting how Fe, Co, and Ni catalysts have been utilized to address the associated challenges for the development of alkyne dihydrosilylation reactions.

## Fe-catalyzed dihydrosilylation of alkynes

**SCHEME 2:**
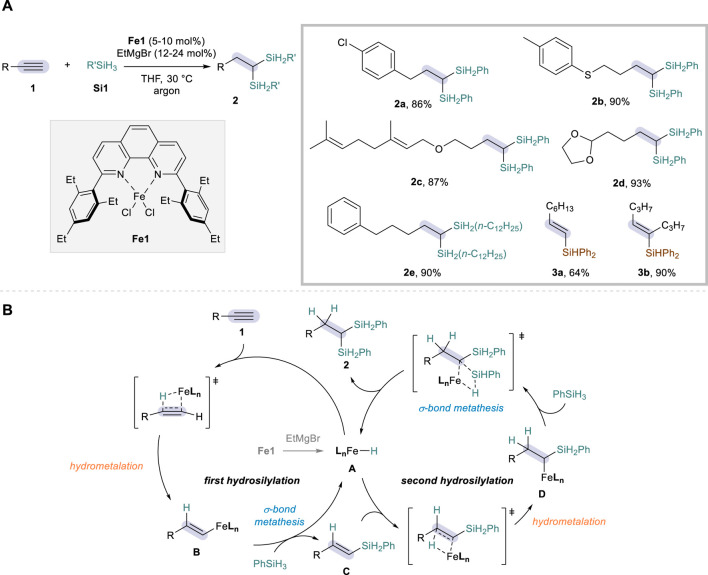
Fe-catalyzed dihydrosilylation of alkynes: **(A)** Selected examples of reaction scope. **(B)** Proposed mechanism.

Iron offers prominence as an ideal catalyst for sustainable chemical reactions, benefiting from its abundance, low costs, and low toxicity (Bauer and Knölker, 2015; Fürstner, 2016). Dihydrosilylation of alkynes, if combined with Fe catalysis, would thus represent a powerful method for the access to disilylalkanes with an excellent atom economy. While Fe-catalyzed protocols have been broadly investigated for monohydrosilylation of alkynes (Greenhalgh et al., 2014; Challinor et al., 2016; Docherty et al., 2017; Hu et al., 2020), the dihydrosilylation congener is less common and the only example was reported by Zhu in 2019 (Hu et al., 2019). Such a development was achieved by employing an iron catalyst bearing 2,9-diaryl-1,10-phenanthroline ligand (**Fe1**), in combination with EtMgBr as an activator, PhSiH_3_ as a hydrosilylation agent, and THF solvent to allow for the conversion of a wide range of aliphatic alkynes **1** (Scheme 2A). In this system, the reaction was proven to be highly efficient and regioselective, furnishing 1,1-disilylalkanes **2** through anti-Markovnikov hydrosilylation followed by the second addition at the carbon α to the silyl group of vinylsilane intermediates. A number of functional groups were tolerated on the terminal alkyne substrates, including aryl chloride (**2a**), sulfide (**2b**), ether (**2c**), acetal (**2d**), and an alkyl silane (*n*-C_12_H_25_SiH_3_) could also be employed as an alternative hydrosilylation source (**2e**). The reaction with a secondary silane (Ph_2_SiH_2_) only led to the corresponding monohydrosilylation products (**3a-b**).

The reaction was proposed to proceed via activation of Fe(II) to a low-valent Fe(I) species **A** with EtMgBr playing a role as the reductant, after which the catalytic cycle kicks off to promote the hydrosilylation reactions (Scheme 2B). Based on the precedents of Fe-catalyzed alkene hydrosilylation (Hu et al., 2018), the authors proposed that the reaction takes place through sequential hydrosilylations involving hydrometalation (**1** to **B** & **C** to **D**) and σ-bond metathesis (**B** to **C** & **D** to **2**). The excellent regioselectivity in the second hydrosilylation was attributed to the α-silicon effect of vinylsilane intermediate **C**, which stabilizes the insertion of electropositive metal at the α-position that holds a higher charge density. This mechanism proposal was supported by control experiments showing the rapid formation of (*E*)-vinylsilanes **C** within 10 min under the standard conditions, as well as by the successful conversion of such an intermediate to the dihydrosilylation products **2**. Deuterium labeling experiments further showed that hydrogen is specifically delivered from the silane source.

## Co-catalyzed dihydrosilylation of alkynes

**SCHEME 3:**
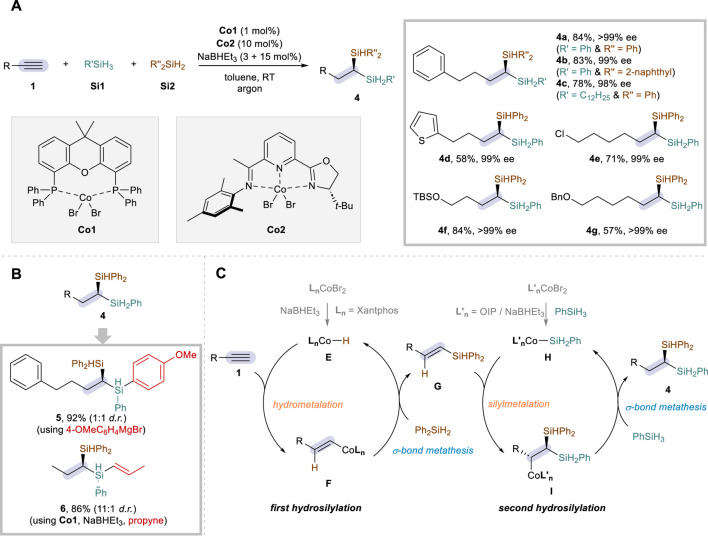
Co-catalyzed enantioselective dihydrosilylation of aliphatic alkynes via dual catalysis. **(A)** Selected examples of reaction scope. **(B)** Synthetic application. **(C)** Proposed mechanism.

It is not exaggerated to state that the development of alkyne dihydrosilylation has been mostly dominated by cobalt catalysis, since the recent advances have seen the advent of a much wider spectrum of dihydrosilylation transformations based on the use of such an earth-abundant metal. In particular, Lu and co-workers led this research field at the forefront, elegantly showing numerous protocols with diverse selectivity. Building upon their previous strategy for the Co-catalyzed alkene hydrosilylation (Cheng et al., 2017), the Lu group reported the first example of Co-catalyzed alkyne dihydrosilylation reactions in 2019 (Guo et al., 2019). The reaction utilizes a dual catalytic system hinging on CoBr_2_·Xantphos (**Co1**) and CoBr_2_·OIP (**Co2**; OIP = oxazoline-iminopyridine), affording 1,1-disilylalkanes (**4**) not only in high efficiency and regioselectivity but also in excellent enantioselectivity (Scheme 3A). Similar to the above-mentioned Fe strategy, additive amounts of a reductant (NaBHEt_3_) were required to activate the Co catalysts, and a variety of primary (**Si1**) and secondary silanes (**Si2**) could be employed together as the silane sources for the sequential additions (**4a**–**4c**). The scope was broad with respect to the aliphatic alkynes (**1**), tolerating a range of functional groups such as heterocyclic motif (**4d**), chloro (**4e**), silyl ether (**4f**), and ether (**4g**) to achieve the products in excellent enantioselectivity. The synthetic virtues of this protocol were proven by the compatibility for gram-scale reactions and further by the site-selective post-modification of the secondary silane moiety of product **4** (Scheme 3B). For instance, the reaction with (4-methoxyphenyl)magnesium bromide gave product **5** via arylation at the secondary silane motif, while the use of **Co1** catalyst in further hydrosilylation with propyne furnished product **6** in a good diastereoselectivity.

The reaction was proposed to operate through two distinctive catalytic cycles, and control experiments corroborated that secondary silane **Si2** is inserted first and CoBr_2_·Xantphos (**Co1**) is essential for this process. Notably, NaBHEt_3_ was suggested to react with **Co1** to form a CoH species **E**, which then joins the first catalytic cycle to enable the hydrosilylation of aliphatic alkyne **1** with **Si2** via hydrometalation and σ-bond metathesis of **F** to form the C–Si bond (Scheme 3C). On the other hand, the second catalytic cycle was proposed to proceed with OIP-based Co–Si species **H** (Wang et al., 2017), allowing for the asymmetric hydrosilylation of vinylsilane **G** with primary silane **Si1** through silylmetalation followed by C–H bond formation. The proposed mechanism was in good agreement with DFT calculations, and the regioselectivity of the second hydrosilylation was elucidated to be directed predominantly by the electronic effects of the silyl group of **G**.

**SCHEME 4:**
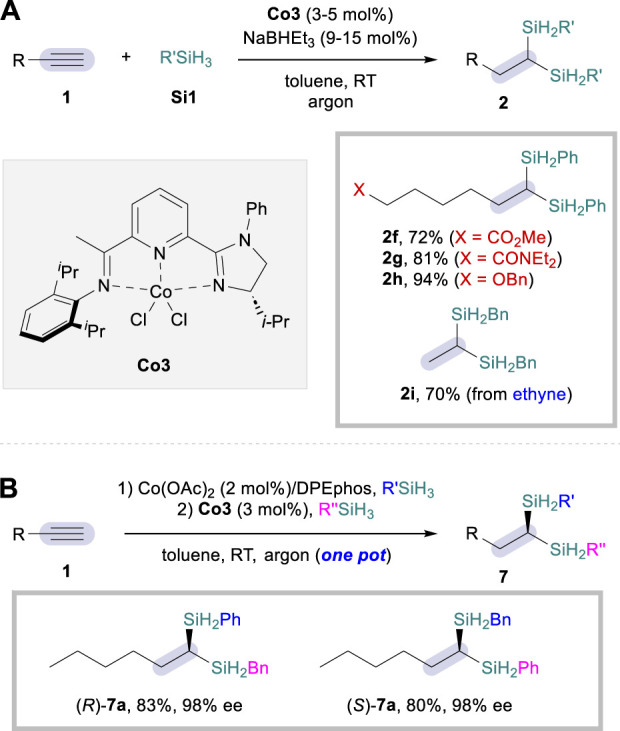
Co-catalyzed dihydrosilylation of aliphatic alkynes. **(A)** Single catalysis system. **(B)** Enantioselective one-pot sequential procedure.

Based on the same mechanistic platform, the Lu group further improved the dihydrosilylation reaction to a single catalytic system (Cheng et al., 2019) that relies on CoCl_2_·IIP complex (**Co3**; IIP = imidazoline iminopyridine), once again facilitating the dihydrosilylation of aliphatic alkynes **1** but rather with primary silanes **Si1** as a sole hydrosilylation reagent (Scheme 4). An enhanced functional group tolerance was observed when compared to the dual catalytic strategy, leading to the corresponding 1,1-disilylalkanes **2** in excellent selectivity and improved efficiency with reduced catalyst loadings (**2f**–**2h**, Scheme 4A). Remarkably, the reaction was also effective for the incorporation of ethyne as the alkyne coupling partner, successfully transforming the gaseous reactant to the corresponding 1,1-disilylalkane product **2i**. Furthermore, the asymmetric sequential dihydrosilylation was also possible by employing a one-pot procedure (Scheme 4B). For instance, both enantiomers of 1,1-disilylalkane **7a** could be obtained by simply changing the addition sequence of the two silane sources under identical reaction conditions. Although this protocol was mechanistically drawn upon the previous dual catalytic system, the authors suggested that the second hydrosilylation step could also proceed via CoH-based hydrometalation/σ-bond metathesis.

**SCHEME 5:**
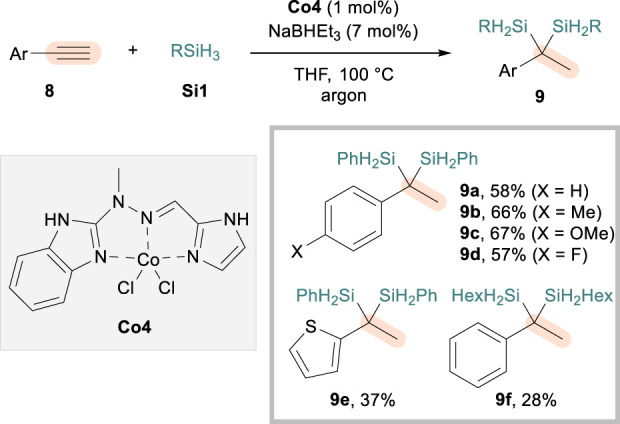
Co-catalyzed dihydrosilylation of aryl alkynes.

While proven to be highly efficient for the access to 1,1-disilylalkanes, the aforementioned protocols were limited to the dihydrosilylation of aliphatic terminal alkynes. The use of rare-earth La (Chen et al., 2020) or borane catalysts (Wang et al., 2021) provided alternative strategies that render such a limitation mitigated to certain extents, furnishing geminal disilylalkanes from aryl alkynes via initial Markovnikov or anti-Markovnikov hydrosilylation, respectively. In 2022, Banach et al. (2022) demonstrated that Co catalysts are also effective for the dihydrosilylation of alkynes bearing an aryl substituent (**8**), albeit requiring elevated reaction temperatures. The initial screening involved the use of Fe catalysts and Fe/Co dual conditions, but **Co4** with an *N*,*N*,*N*-tridentate hydrazone ligand was shown to be most efficient for the reaction employing **Si1**, NaHBEt_3_, and THF solvent (Scheme 5). A range of aryl alkynes could be reacted through sequential Markovnikov-selective hydrosilylations, furnishing geminal aryl (disilyl)alkanes **9** in excellent regioselectivity but with moderate efficiency. Common substituents such as methyl (**9b**), methoxy (**9c**), and fluoro (**9d**) could be tolerated, and so were thiophenyl alkyne (**9e**) and aliphatic silane (**9f**). While the mechanistic picture was not drawn in detail, the authors utilized ^1^H and ^11^B NMR experiments to suggest that NaHBEt_3_ participates not only as a reductant for the formation of CoH species but also as a base to modify the ligand and influence the catalytic activity of the metal center.

**SCHEME 6:**
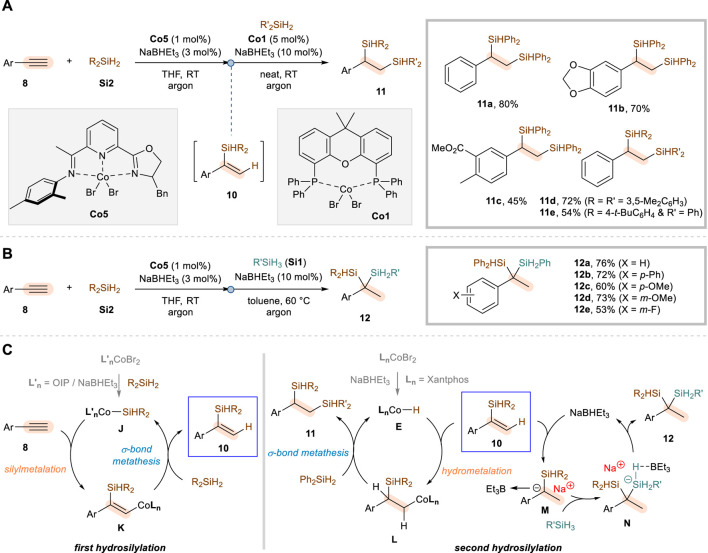
Co-catalyzed regiodivergent dihydrosilylation of aryl alkynes. **(A)** α,β-selective dihydrosilylation. **(B)** α,α-selective dihydrosilylation. **(C)** Proposed mechanism.

In 2023, Lu and co-workers reported a novel method to selectively achieve not only α,α- but also α,β-dihydrosilylation of aryl alkynes (Cheng et al., 2023). These regiodivergent reactions commonly employ NaBHEt_3_ as an activator, CoBr_2_·OIP (**Co5**) as a precatalyst, secondary silanes **Si2**, and THF solvent for the first hydrosilylation step in reaction with aryl alkynes **8**, but differed reaction conditions are needed to diversify the regioselectivity of the second hydrosilylation of α-substituted vinylsilane intermediates **10** (Scheme 6). The α,β-dihydrosilylation was achieved by utilizing CoBr_2_·Xantphos (**Co1**) under neat conditions in the second addition step, which led to the vicinal disilylalkanes **11** via anti-Markovnikov hydrosilylation of vinylsilane intermediates **10** with Ph_2_SiH_2_ (Scheme 6A). Under these reaction conditions, a variety of aryl alkynes were reacted for the α,β-dihydrosilylation, showing a good functional group tolerance (**11a**–**11c**) and compatibility with other secondary silane sources (**11d**–**11e**). On the other hand, the α,α-dihydrosilylation was realized by further addition of NaBHEt_3_, in combination with toluene solvent and primary silanes **Si1** as an alternative second hydrosilylation reagent since the sterically more encumbered secondary silanes hinder the incorporation of silyl groups at the same carbon (Scheme 6B). A range of aryl alkynes **8** could be transformed to the corresponding geminal disilylalkanes **12**, with a similar level of functional group tolerance in comparison to the α,β-dihydrosilylation system.

For the initial α-selective hydrosilylation of terminal aryl alkynes, the catalysis involving Co–Si species was proposed in which the migratory insertion of the alkyne into the Co–Si bond (**J** to **K**) is followed by σ-bond metathesis (**K** to **10**) with a secondary silane (Scheme 6C). The succeeding anti-Markovnikov hydrosilylation leading to α,β-dihydrosilylation products **11** was then proposed to involve a CoH species **E**, which is initially generated from the reaction between CoBr_2_·Xantphos (**Co1**) and NaBHEt_3_. The hydrometalation of the α-substituted vinylsilane intermediate **10** proceeds with a selective insertion of the cobalt at the β-position (**L**), thus eventually furnishing the vicinal bis(silane) products **11** through C–Si bond-forming σ-bond metathesis. In contrast, the authors proposed that the second hydrosilylation leading to α,α-dihydrosilylation products **12** is rather catalyzed by NaBHEt_3_, generating α-silyl-α-phenylcarbanion **M** through a hydride transfer. It was presumed that coordination by triethylboron and the subsequent reaction with a primary silane (**N**) would then follow to give the geminal bis(silane) products **12** and regenerate NaBHEt_3_.

## Ni-catalyzed dihydrosilylation of alkynes

**SCHEME 7:**
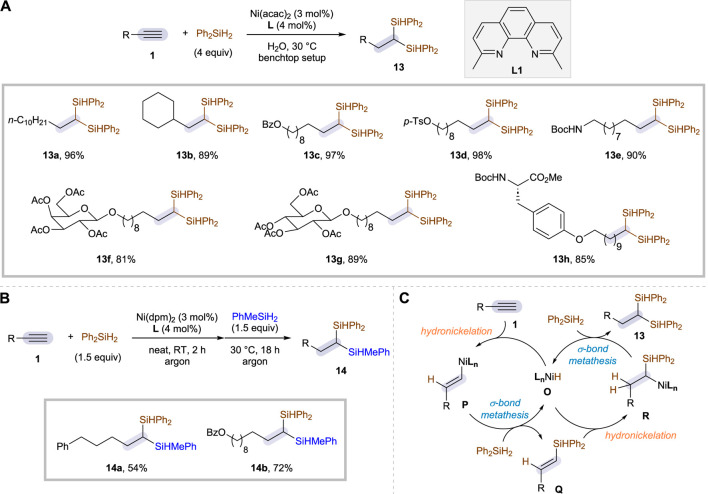
Ni-catalyzed dihydrosilylation of aliphatic alkynes in aqueous and aerobic conditions. **(A)** Selected examples of reaction scope. **(B)** Sequential dihydrosilylation. **(C)** Proposed mechanism.

One of the common requirements observed in most of the earth-abundant metal-catalyzed alkyne dihydrosilylation strategies is the need of an air- and moisture-sensitive reductant to activate the precatalyst into a low-valent catalytic species. Such a prerequisite, as a corollary, lowers the synthetic maneuverability owing to the consequent need of reaction set-up under an inert atmosphere. In this regard, Ni is a good candidate as a potential catalyst that could preclude the use of strong reductants, since the formation of low-valent Ni species is well precedented even with silanes as a mild reductant (Eberhardt and Guan, 2016). Nonetheless, Ni-catalyzed hydrosilylation of alkynes is relatively rare, and the few reported examples of monohydrosilylation reactions are mainly based on the use of a porous organic polymer (POP) as a heterogeneous ligand (Zhou et al., 2018). Very recently, Jung and co-workers reported a Ni-catalyzed protocol for dihydrosilylation of alkynes, a highly practical method that circumvents the need of strong reductants while being operative even under aqueous and aerobic conditions (Lee et al., 2024). The reaction was found to be highly effective using Ni(acac)_2_ as a precatalyst, 2,9-dimethyl-1,10-phenanthroline ligand **L1**, Ph_2_SiH_2_ as the sole silane source, and H_2_O solvent at 30°C, resulting in the formation of 1,1-disilylalkanes **13** with excellent regioselectivity (Scheme 7A). Although the reaction was also compatible with organic solvents or under neat conditions, the reaction rate was found to be escalated when H_2_O solvent was employed.

The substrate scope is broad and includes not only simple aliphatic alkynes (**13a**–**13e**) but also those substituted with a biologically relevant moiety (**13f**–**13h**): galactose, glucose, and tyrosine derivatives could all be incorporated successfully. Moreover, the installations of two distinctive silyl groups were possible through sequential additions of Ph_2_SiH_2_ and Ph(Me)SiH_2_, affording the corresponding 1,1-disilylalkane products **14** in good yields (Scheme 7B). A range of functional groups could be tolerated once again, but the attempts for asymmetric induction with chiral ligands were unsuccessful. It is worthwhile to mention that 1,1-addition of silanes is generally challenging if both silane sources being inserted are secondary silanes. Indeed, a primary silane was required as the second silane reagent for the previous reactions leading to 1,1-disilylalkanes, enabling the transformation by easing the steric effects. In contrast, the Ni system was shown to be surprisingly efficient for the geminal insertions of two secondary silanes. The authors conducted a series of control experiments and DFT calculations, and subsequently proposed a mechanism consisting of two identical catalytic cycles, one catalyzing the hydrosilylation of alkyne **1** and the other one promoting the conversion of (*E*)-vinylsilane intermediate **Q** (Scheme 7C). The precatalyst was suggested to be converted to NiH species **O** as an active catalyst when treated with Ph_2_SiH_2_, which then undergoes a series of hydronickelation (**1** to **P** and **Q** to **R**) followed by C–Si bond-forming σ-bond metathesis (**P** to **Q** and **R** to **13**). This operation mode was computed to be consistent with the observed regioselectivity.

## Conclusion and outlook

In this mini-review, we have highlighted recent developments of earth-abundant transition metal-catalyzed strategies for dihydrosilylation of alkynes. Given the significant electronic and steric differences between alkynes and vinylsilanes, monohydrosilylation is more common with alkynes, as the second addition would necessitate differed catalytic conditions. Recent advancements have addressed this challenge by either utilizing dual catalyst systems or enhancing the performance of a single catalyst to enable both additions. Catalytic systems based on Fe, Co, and Ni have been reported, each exhibiting distinctive features in terms of substrate compatibility, regioselectivity, practicality, and reaction mechanism. The Fe system has been proven selective for the dihydrosilylation of aliphatic terminal alkynes leading to 1,1-disilylalkanes, utilizing EtMgBr as an activator and PhSiH_3_ as a silane source. The Co systems are more diverse and have offered enantioselective methods for the synthesis of 1,1-disilylalkanes, as well as regiodivergent strategies for the dihydrosilylation of aryl alkynes, be it based on single or dual catalysts. In the Co systems, NaBHEt_3_ has been commonly employed as an activator and a wider spectrum of silane reagents have been applied. The Ni system has been developed more recently and offers a practical method that operates under atmospheric conditions while circumventing the need of strong reductants. The efficiency is not deteriorated by the presence of air or water in this system, thus rendering the synthetic maneuver extremely simple for access to 1,1-disilylalkanes.

Despite these advancements, this research area is still in its infancy and the reaction development for unseen selectivity is highly desired. For instance, the dihydrosilylation of aliphatic alkynes has been mainly developed for the 1,1-addition and, as such, the investigation toward 1,2- or 2,1- or 2,2-additions could be an interesting research topic, especially for the development of enantioselective reactions. Lu and co-workers showed that the dihydrosilylation of aryl alkynes could be achieved in a regiodivergent manner, and the study toward its asymmetric version would also be of great significance. The reaction efficiency should also be improved for sterically encumbered substrates, including internal alkynes, which have been shown operational only for the monohydrosilylation. Furthermore, there is a demand for detailed investigations of the reaction mechanisms. Two commonly proposed operation modes are: 1) migratory insertion of alkyne or vinylsilane into M–H bond followed by C–Si bond-forming σ-bond metathesis, and 2) migratory insertion of alkyne or vinylsilane into M–Si bond followed by C–H bond-forming σ-bond metathesis. These mechanisms have been proposed mainly based on DFT calculations, kinetic studies, and control experiments, thus lacking evidence that provides more direct insights. The isolation of key intermediates would be an appealing strategy for the sophisticated elucidation of the involved reaction mechanisms.

## References

[B1] Banach Ł. Brykczyńska D. Gorczyński A. Wyrzykiewicz B. Skrodzki M. Pawluć P. (2022). Markovnikov-selective double hydrosilylation of challenging terminal aryl alkynes under cobalt and iron catalysis. Chem. Commun. 58, 13763–13766. 10.1039/D2CC04015H 36421006

[B2] Bauer I. Knölker H.-J. (2015). Iron catalysis in organic synthesis. Chem. Rev. 115, 3170–3387. 10.1021/cr500425u 25751710

[B3] Challinor A. J. Calin M. Nichol G. S. Carter N. B. Thomas S. P. (2016). Amine-activated iron catalysis: air- and moisture-stable alkene and alkyne hydrofunctionalization. Adv. Synth. Catal. 358, 2404–2409. 10.1002/adsc.201600570

[B4] Chen J. Guo J. Lu Z. (2018). Recent advances in hydrometallation of alkenes and alkynes via the first row transition metal catalysis. Chin. J. Chem. 36, 1075–1109. 10.1002/cjoc.201800314

[B5] Chen W. Song H. Li J. Cui C. (2020). Catalytic selective dihydrosilylation of internal alkynes enabled by rare-earth ate complex. Angew. Chem. Int. Ed. 59, 2365–2369. 10.1002/anie.201913773 31793164

[B6] Cheng B. Lu P. Zhang H. Cheng X. Lu Z. (2017). Highly enantioselective cobalt-catalyzed hydrosilylation of alkenes. J. Am. Chem. Soc. 139, 9439–9442. 10.1021/jacs.7b04137 28654260

[B7] Cheng Z. Li M. Zhang X.-Y. Sun Y. Yu Q.-L. Zhang X.-H. (2023). Cobalt-catalyzed regiodivergent double hydrosilylation of arylacetylenes. Angew. Chem. Int. Ed. 62, e202215029. 10.1002/anie.202215029 36330602

[B8] Cheng Z. Xing S. Guo J. Cheng B. Hu L.-F. Zhang X.-H. (2019). Highly regioselective sequential 1,1-dihydrosilylation of terminal aliphatic alkynes with primary silanes. Chin. J. Chem. 37, 457–461. 10.1002/cjoc.201900079

[B9] de Almeida L. D. Wang H. Junge K. Cui X. Beller M. (2021). Recent advances in catalytic hydrosilylations: developments beyond traditional platinum catalysts. Angew. Chem. Int. Ed. 60, 550–565. 10.1002/anie.202008729 PMC7839722 32668079

[B10] Docherty J. H. Peng J. Dominey A. P. Thomas S. P. (2017). Activation and discovery of earth-abundant metal catalysts using sodium *tert*-butoxide. Nat. Chem. 9, 595–600. 10.1038/nchem.2697 28537588

[B11] Eberhardt N. A. Guan H. (2016). Nickel hydride complexes. Chem. Rev. 116, 8373–8426. 10.1021/acs.chemrev.6b00259 27437790

[B12] Fürstner A. (2016). Iron catalysis in organic synthesis: a critical assessment of what it takes to make this base metal a multitasking champion. ACS Cent. Sci. 2, 778–789. 10.1021/acscentsci.6b00272 27981231 PMC5140022

[B13] Greenhalgh M. D. Frank D. J. Thomas S. P. (2014). Iron-Catalysed chemo-regio-and stereoselective hydrosilylation of alkenes and alkynes using a bench-stable iron(II) pre-catalyst. Adv. Synth. Catal. 356, 584–590. 10.1002/adsc.201300827

[B14] Greenhalgh M. D. Jones A. S. Thomas S. P. (2015). Iron-Catalysed hydrofunctionalisation of alkenes and alkynes. ChemCatChem 7, 190–222. 10.1002/cctc.201402693

[B15] Guo J. Wang H. Xing S. Hong X. Lu Z. (2019). Cobalt-catalyzed asymmetric synthesis of gem-Bis(silyl)alkanes by double hydrosilylation of aliphatic terminal alkynes. Chem 5, 881–895. 10.1016/j.chempr.2019.02.001

[B16] He P. Hu M.-Y. Zhang X.-Y. Zhu S.-F. (2022). Transition-metal-catalyzed stereo- and regioselective hydrosilylation of unsymmetrical alkynes. Synthesis 54, 49–66. 10.1055/a-1605-9572

[B17] Hu M.-Y. He P. Qiao T.-Z. Sun W. Li W.-T. Lian J. (2020). Iron-catalyzed regiodivergent alkyne hydrosilylation. J. Am. Chem. Soc. 142, 16894–16902. 10.1021/jacs.0c09083 32945664

[B18] Hu M.-Y. He Q. Fan S.-J. Wang Z.-C. Liu L.-Y. Mu Y.-J. (2018). Ligands with 1,10-phenanthroline scaffold for highly regioselective iron-catalyzed alkene hydrosilylation. Nat. Commun. 9, 221. 10.1038/s41467-017-02472-6 29335560 PMC5768772

[B19] Hu M.-Y. Lian J. Sun W. Qiao T.-Z. Zhu S.-F. (2019). Iron-catalyzed dihydrosilylation of alkynes: efficient access to geminal bis(silanes). J. Am. Chem. Soc. 141, 4579–4583. 10.1021/jacs.9b02127 30810313

[B20] Lee C. Jeon J. H. Lee S. Choe W. Kwak J. Seo S. (2024). Nickel-catalyzed mono- and dihydrosilylation of aliphatic alkynes in aqueous and aerobic conditions. ACS Catal. 14, 5077–5087. 10.1021/acscatal.4c00109

[B21] Marciniec B. (2009) Hydrosilylation: a comprehensive review on recent advances. Dordrecht: Springer.

[B22] Nakajima Y. Shimada S. (2015). Hydrosilylation reaction of olefins: recent advances and perspectives. RSC Adv. 5, 20603–20616. 10.1039/C4RA17281G

[B23] Obligacion J. V. Chirik P. J. (2018). Earth-abundant transition metal catalysts for alkene hydrosilylation and hydroboration. Nat. Rev. Chem. 2, 15–34. 10.1038/s41570-018-0001-2 30740530 PMC6365001

[B24] Roesky H. W. (2017) Efficient methods for preparing silicon compounds. Boston: Academic Press.

[B25] Shimada T. Mukaide K. Shinohara A. Han J. W. Hayashi T. (2002). Asymmetric synthesis of 1-Aryl-1,2-ethanediols from arylacetylenes by palladium-catalyzed asymmetric hydrosilylation as a key step. J. Am. Chem. Soc. 124, 1584–1585. 10.1021/ja017617g 11853426

[B26] Sun J. Deng L. (2016). Cobalt complex-catalyzed hydrosilylation of alkenes and alkynes. ACS Catal. 6, 290–300. 10.1021/acscatal.5b02308

[B27] Wang C. Teo W. J. Ge S. (2017). Cobalt-catalyzed regiodivergent hydrosilylation of vinylarenes and aliphatic alkenes: ligand- and silane-dependent regioselectivities. ACS Catal. 7, 855–863. 10.1021/acscatal.6b02518

[B28] Wang G. Su X. Gao L. Liu X. Li G. Li S. (2021). Borane-catalyzed selective dihydrosilylation of terminal alkynes: reaction development and mechanistic insight. Chem. Sci. 12, 10883–10892. 10.1039/D1SC02769G 34476068 PMC8372554

[B29] Zaranek M. Marciniec B. Pawluć P. (2016). Ruthenium-catalysed hydrosilylation of carbon–carbon multiple bonds. Org. Chem. Front. 3, 1337–1344. 10.1039/C6QO00261G

[B30] Zhou Y.-B. Liu Z.-K. Fan X.-Y. Li R.-H. Zhang G.-L. Chen L. (2018). Porous organic polymer as a heterogeneous ligand for highly regio- and stereoselective nickel-catalyzed hydrosilylation of alkyne. Org. Lett. 20, 7748–7752. 10.1021/acs.orglett.8b03064 30495967

